# Recurrent diabetic myonecrosis in an African American woman with long-standing uncontrolled type 2 diabetes: a case report

**DOI:** 10.1186/s13256-023-03896-z

**Published:** 2023-06-13

**Authors:** Varsha Kurup, Ying Nagoshi, Marie Rivera-Zengotita, Edlira Maska

**Affiliations:** 1 Division of General Internal Medicine, Department of Internal Medicine, University of Florida College of Medicine, PO Box 100277, Gainesville, FL 32610 USA; 2 Department of Radiology, University of Florida College of Medicine, Gainesville, FL USA

**Keywords:** Diabetes mellitus, Recurrent diabetic myonecrosis, Asymmetric leg swelling, Diabetic muscle infarction, Case report

## Abstract

**Background:**

Diabetic myonecrosis, also called diabetic muscle infarction, is an uncommon complication of uncontrolled diabetes mellitus and is frequently underdiagnosed. The objective of this case report is to highlight the challenges in the early diagnosis and treatment of this disease.

**Case presentation:**

A 51-year-old African American woman with a long history of uncontrolled diabetes mellitus presented to her primary care physician with right thigh pain. A diagnosis of diabetes myonecrosis was made on the basis of magnetic resonance imaging, biopsy, and negative autoimmune panel. After failing conservative treatment, the patient was treated with prednisone with gradual improvement of her symptoms. However, she had a recurrence of myonecrosis almost one year after her original presentation, which was also treated with prednisone. The recurrence had a shorter course and the patient recovered well. Challenges to the treatment in this patient were her debilitating pain and her underlying chronic kidney disease.

**Conclusions:**

A high index of suspicion for diabetic myonecrosis is necessary when a patient with diabetes presents with unilateral focal leg pain and swelling. Magnetic resonance imaging and biopsy can help confirm the diagnosis. Prednisone may be considered in patients who lack spontaneous regression with just rest. Educating healthcare professionals about this uncommon condition is of utmost importance in avoiding unnecessary testing and inappropriate treatment.

## Background

It is common for patients with poorly controlled type 1 or type 2 diabetes to develop micro- and macrovascular complications such as retinopathy, nephropathy, and neuropathy. Diabetic myonecrosis is an uncommon complication, therefore frequently not recognized and treated in a timely manner. In a patient with long-standing diabetes with microvascular complications, sudden onset of acute atraumatic focal limb pain should prompt addition of diabetic myonecrosis to the differential. Diabetic myonecrosis is ischemic necrosis of skeletal muscle; the pathogenesis is currently not understood [1]. Even when the diagnosis is confirmed, management remains a challenge since there are no evidence-based guidelines, and the treatment options are limited by the patients’ comorbidity.

Here we describe the diagnosis and management of a patient who presented with focal right leg swelling and pain.

## Case presentation

**Fig. 1:**
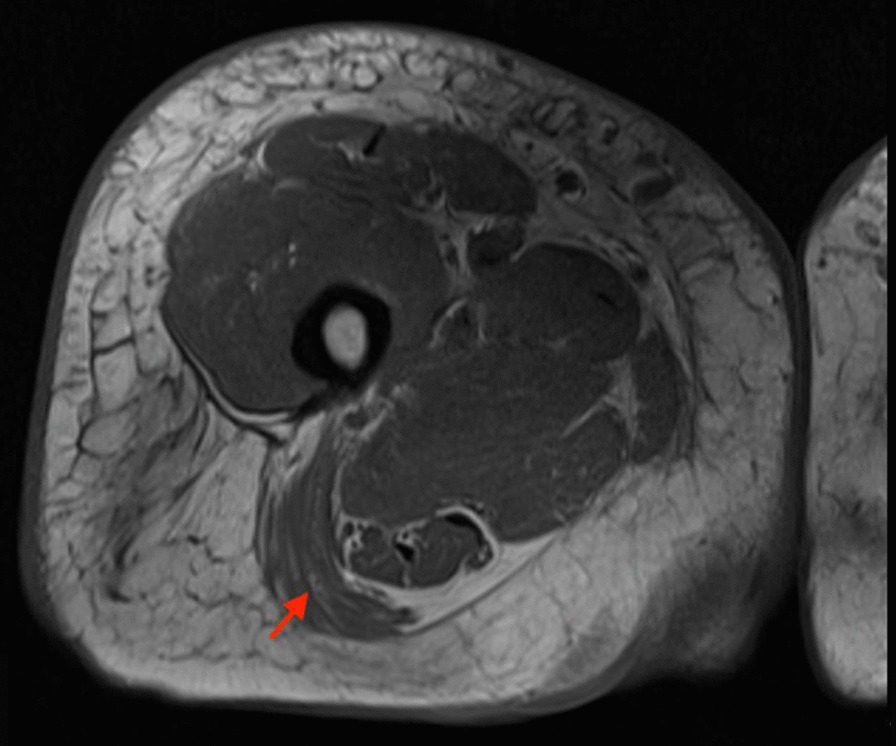
MRI right thigh with arrow showing perimysial edema surrounding the biceps femoris muscles

**Fig. 2:**
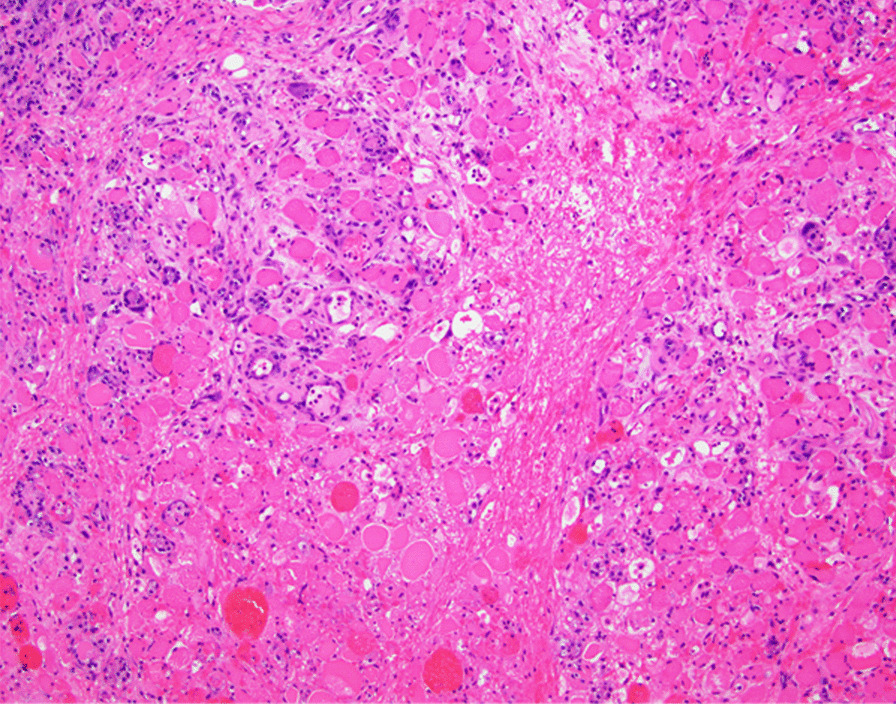
The skeletal muscle biopsy shows myopathic features, including marked variation in myofiber diameter with frequent small rounded myofibers, endomysial fibrosis, and increased internal nuclei. Hematoxylin and eosin (H&E), ×100

**Fig. 3:**
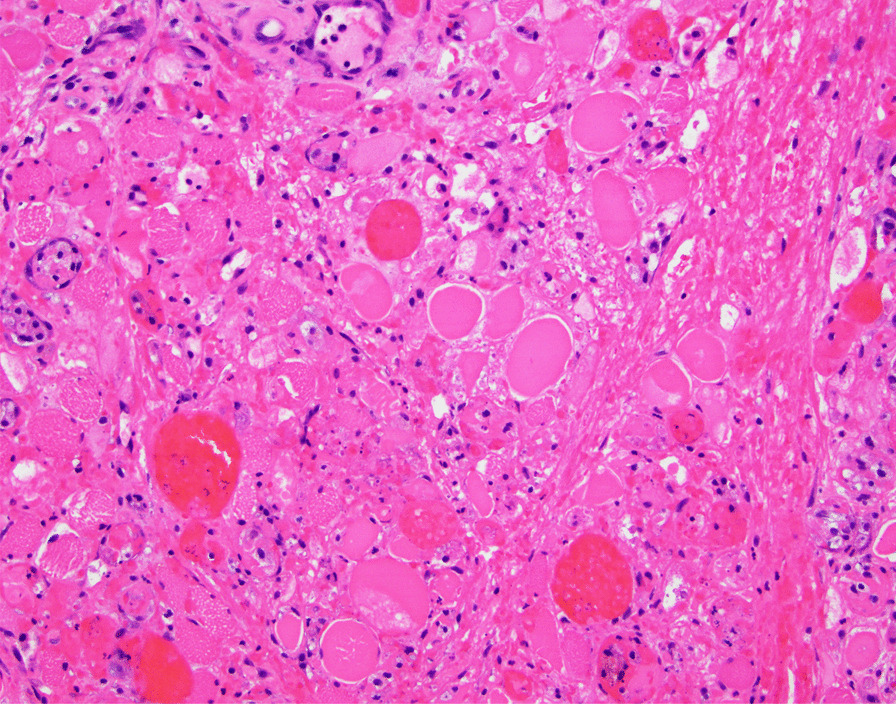
Numerous necrotic myofibers are identified. H&E, ×200

**Fig. 4:**
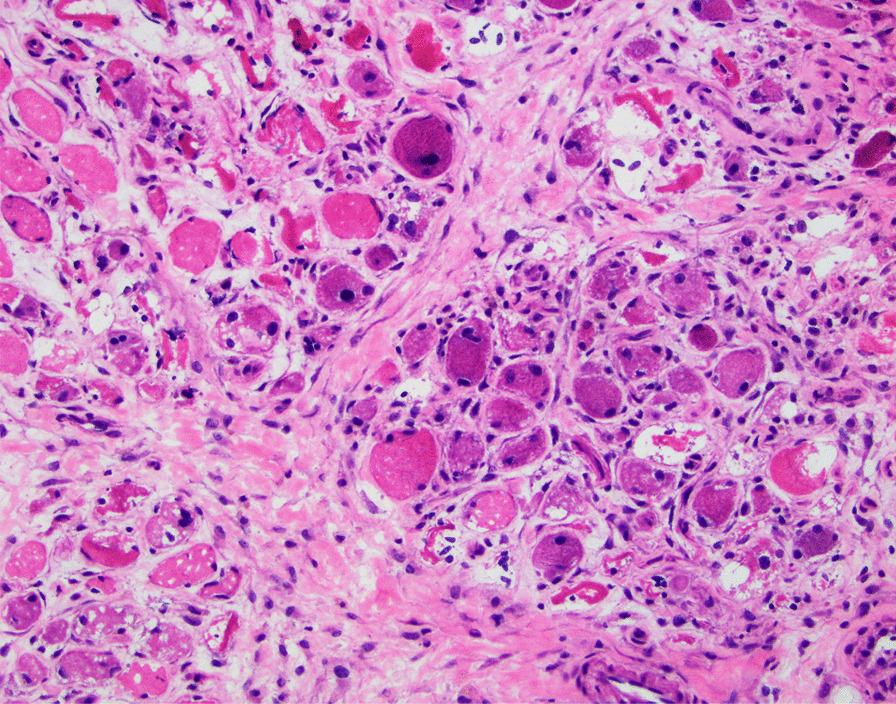
Basophilic myofibers with large nuclei and prominent nucleoli consistent with regenerating fibers. H&E, ×200

**Fig. 5:**
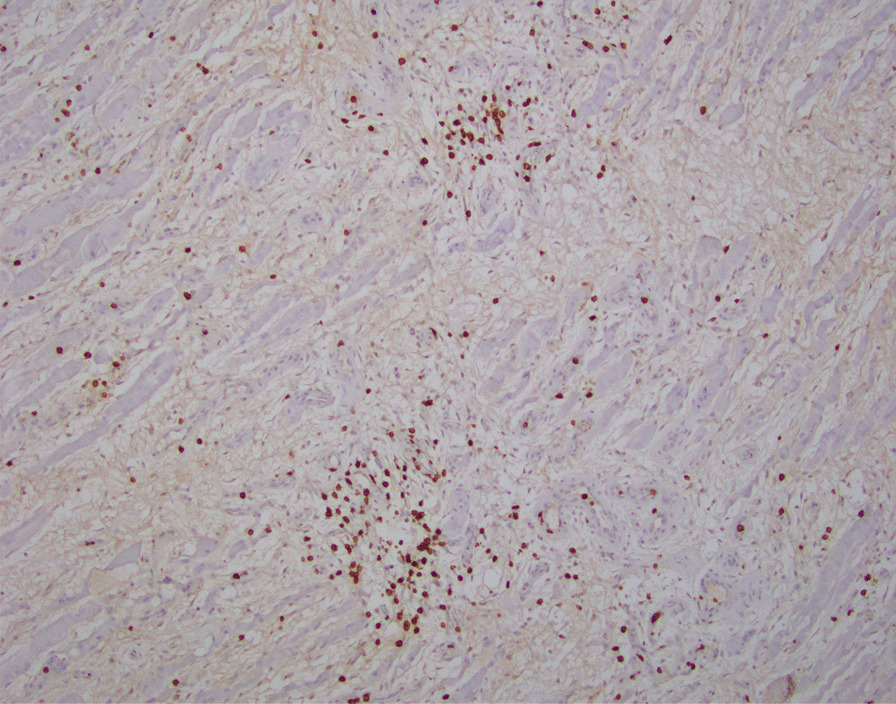
Endomysial and perivascular aggregates of T lymphocytes are seen. CD3 immunostudy, ×200

**Fig. 6:**
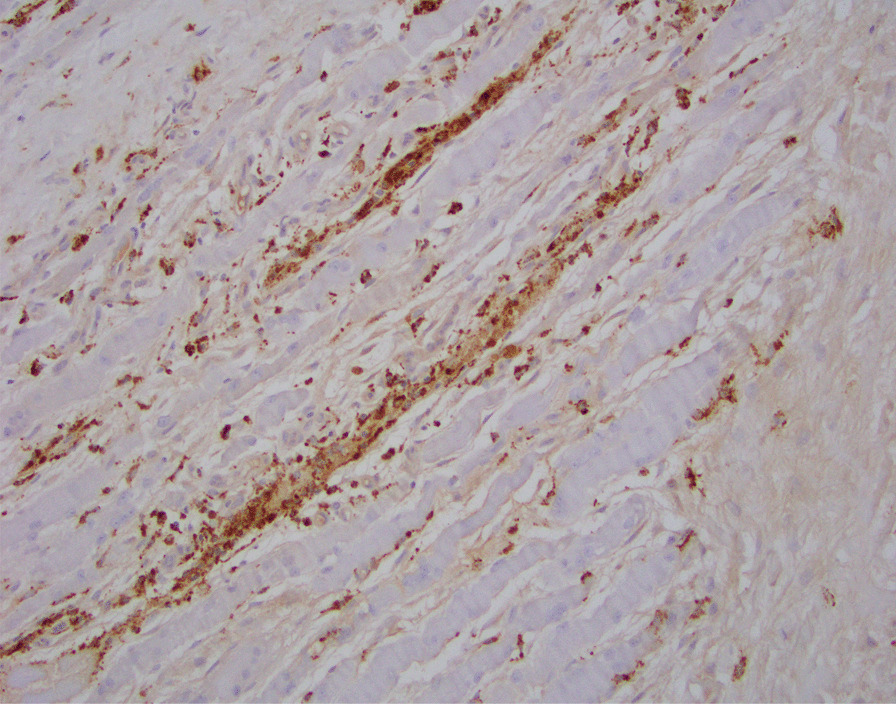
Immunostudy for CD68 shows numerous perivascular and endomysial macrophages, as well as macrophages invading myofibers (myophagocytosis). CD68 immunostudy, ×200

A 51-year-old African American woman presented to the internal medicine clinic with 2-week duration of right posterior medial thigh swelling and pain. She had a 20-year history of type 2 diabetes with diabetic retinopathy and diabetic nephropathy stage 4. She had no fever or trauma. On physical examination her right thigh had a focal swelling and tenderness mostly to the posterior medial aspect of the thigh. Her dorsalis pedis and posterior tibialis pulses were palpable bilaterally. Her strength and sensation were intact. Deep venous thrombosis was ruled out with duplex ultrasound at emergency department 4 days prior to presentation. Her laboratory results showed hemoglobin A1C 9%, white blood cell count (WBC) 4.2 × 10^3^/μl (4–10 × 10^3^/μl), erythrocyte sedimentation rate (ESR) 120 mm/hour (0–10 mm/hour), high sensitivity C-reactive protein (hs-CRP) 68.51 mg/L (0–5 mg/L), and serum creatinine 2.4 mg/dL (0.51–1.18 mg/dL). Magnetic resonance imaging (MRI) of the right thigh showed patchy muscular enhancement, most confluent in the biceps femoris (Fig. 1). The imaging was concerning for subacute myonecrosis versus asymmetric inflammatory myopathy versus chronic compartment syndrome. She underwent open biopsy of the right thigh, which showed marked variation in myofiber diameter with frequent small rounded myofibers, endomysial fibrosis, and increased internal nuclei (Fig. 2). There were numerous necrotic myofibers, basophilic myofibers with large nuclei and prominent nucleoli consistent with regenerating fibers, and endomysial and perivascular aggregates of T lymphocytes (Figs. 3, 4, 5). Immunostudy for CD68 shows numerous perivascular and endomysial macrophages, as well as macrophages invading myofibers (myophagocytosis) (Fig. 6). Pathological diagnosis was myofiber necrosis. She was then seen by rheumatology. Antinuclear antibodies (ANA), aldolase, creatinine kinase (CK), and myositis panel, which includes Jo-1 Ab, PL-7 Ab, PL-12 Ab, EJ Ab, OJ Ab, SRP Ab, Mi-2 Alpha Ab, Mi-2 Beta Ab, MDA5 Ab, TIF1 Gamma Ab, and NXP-2 Ab, were unremarkable. An electromyography (EMG) showed myopathic changes of the right vastus lateralis, consistent with focal myositis. This EMG sufficiently ruled out diabetic amyotrophy, as she did not experience muscle weakness, nor did she have evidence of patchy involvement of sensory, motor, and autonomic nerves. On the basis of her history and diagnostic findings, clinical diagnosis of diabetic myonecrosis was made.

Patient was on low-dose aspirin, chronically. With her underlying chronic kidney disease, nonsteroidal antiinflammatory drugs were not given. Due to lack of spontaneous regression, 2 months after her initial presentation, she was placed on prednisone 20 mg daily for 1 month. Her symptoms gradually resolved.

**Fig. 7:**
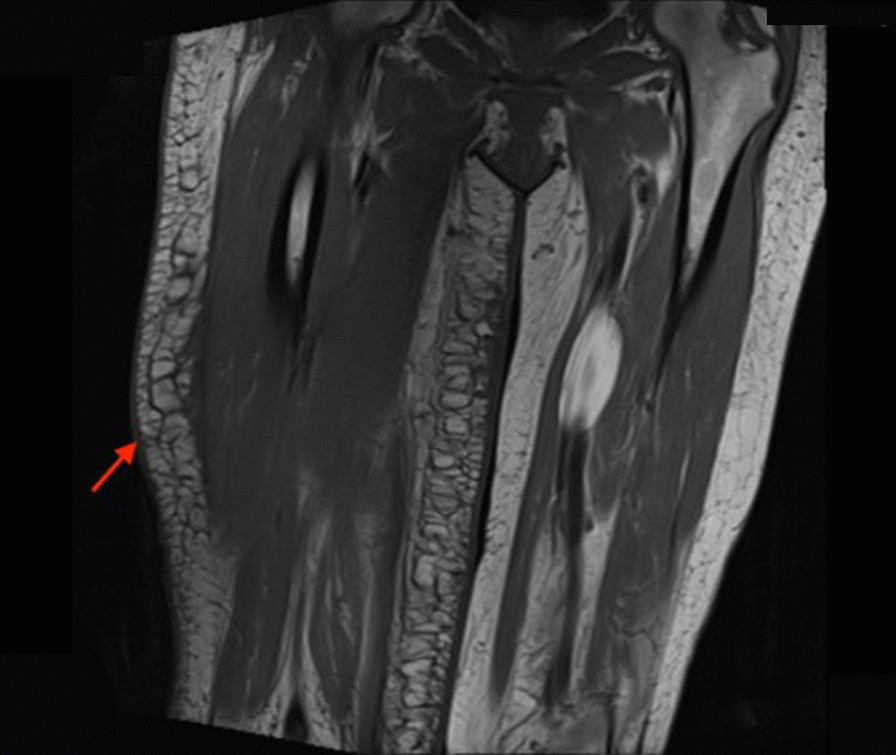
MRI right calf with arrow showing asymmetric increased edema within right thigh hypodermis

However, about 1 year after her initial presentation, she returned to the clinic with the complaint of persistent right calf pain. Due to concern of recurrence of her diabetic myonecrosis, MRI was done, which showed unilateral muscular edema involving right calf with the greatest degree of involvement within medial soleus (Fig. 7). At that time, her hemoglobin A1C was noted to be 8.6%. The patient was diagnosed with having a recurrence of diabetes myonecrosis. Oral prednisone was restarted. Her symptoms resolved within 1 month. It has been over one year since her second course of treatment. She has not had any further recurrence. Patient voiced satisfaction about the treatment she received for her diabetic myonecrosis. She noticed no immediate side effect with steroid use.

## Discussion and conclusions

When a patient presents with focal atraumatic limb pain, differential diagnosis should include deep vein thrombosis, malignant tumor with central necrosis, diabetic amyotrophy, focal myositis, and pyomyositis. Compartment syndrome should be ruled out. If the patient also has long-standing, uncontrolled type 1 or type 2 diabetes, particularly with evidence of end-stage organ damage, diabetic myonecrosis (in other words diabetic muscle infarction) should be considered.

Because diabetic myonecrosis is sufficiently uncommon, most physicians have not seen it. Diabetic myonecrosis was first described by Angervall and Stener [1]. Myonecrosis refers to ischemic necrosis of skeletal muscle unrelated to arterial occlusion or atheroembolism. Pathophysiology is currently poorly understood.

Literature review of multiple case reports revealed that myonecrosis is a rare complication of a poorly controlled diabetes, as evidenced by elevated hemoglobin A1C levels, as well as advanced diabetes with multiple system involvement. In a 2015 review of 126 cases of diabetic myonecrosis, the average hemoglobin A1C at the time of myonecrosis presentation was 9.34%. Patients who presented with the disease also had an increased incidence of at least two other complications of diabetes, the most common being nephropathy [2].

It is important to be familiar with the common clinical presentation, in addition to risk factors for development. The most common symptoms of diabetic myonecrosis are sudden onset of pain in a large muscle group, along with localized swelling. The proximal leg is commonly affected with symptoms commonly occurring unilaterally [2]. Recurrence may occur and usually in a different location. In our patient, the initial occurrence was at right biceps femoris, with recurrence at right medial soleus.

Diagnosis of diabetic myonecrosis is usually made on the basis of MRI findings, with or without biopsy [2]. Diabetic myonecrosis is especially difficult to differentiate from focal myositis. MRI in focal myositis usually shows evidence of contrast-enhanced solitary mass or hypertrophy of a single muscle, with adjacent structures remaining unaffected. Diabetic myonecrosis, on MRI, most commonly shows myositis, as well as necrosis, with surrounding soft tissue edema [3] as it was observed in our patient’s case. Primary pathological findings from the biopsy include edema and necrosis in diabetic myonecrosis. Arteriole and capillary occlusion by fibrin [4, 5] is sometimes seen, but was not in the case of our patient. As part of our patient’s workup, autoimmune and myositis panel were also done after the biopsy and the results were negative.

There is no standardized treatment for diabetic myonecrosis. In multiple case reports, care has usually involved nonsteroidal antiinflammatory analgesics and initial bed rest. However, nonsteroidal antiinflammatory analgesic treatment can worsen chronic kidney disease, which is often seen in these patients. Therefore, most patients with chronic kidney disease were treated instead with an analgesic (acetaminophen, opioids), bed rest, and optimization of glycemic control [2]. Self-limited regression of diabetic myonecrosis is common within a few weeks, but steroids can be utilized for treatment in patients with persistent symptoms [6].

All in all, diabetic myonecrosis may be easily misdiagnosed in a primary care setting as it is a rare complication of diabetes mellitus. Educating primary care physicians and other healthcare providers about its presentation may help avoid unnecessary diagnostic tests and inappropriate treatments. We believe that routinely including diabetic myonecrosis in the discussion of diabetic complications, and developing clear treatment guidelines, will lead to better recognition of diabetic myonecrosis by primary care physicians, as well as improve treatment outcomes for our patients.

## Data Availability

Patient’s electronic medical records are available for review if needed.

## Abbreviations

WBC: White blood cell
ESR: Erythrocyte sedimentation rate
hs-CRP: High sensitivity C-reactive protein
MRI: Magnetic resonance imaging
CD68: Cluster of differentiation 68
ANA: Antinuclear antibodies
CK: Creatinine kinase
