# Correction: Recurrent diabetic myonecrosis in an African American woman with long-standing uncontrolled type 2 diabetes: a case report

**DOI:** 10.1186/s13256-023-04077-8

**Published:** 2023-08-02

**Authors:** Varsha Kurup, Ying Nagoshi, Marie Rivera‑Zengotita, Edlira Maska

**Affiliations:** 1 Division of General Internal Medicine, Department of Internal Medicine, University of Florida College of Medicine, PO Box 100277, Gainesville, FL 32610 USA; 2 Department of Radiology, University of Florida College of Medicine, Gainesville, FL USA

**Correction** **:** **Journal of Medical Case Reports (2023) 17: 271** **https://doi.org/10.1186/s13256-023-03896-z**

Following publication of the original article [1], the authors identified an error in the author name of Edlira Maska.

The incorrect author name is: Adlira Maska.

The correct author name is: Edlira Maska.

The author group has been updated above and the original article [1] has been corrected.
